# Solid Subtype of Papillary Thyroid Carcinoma: A Case Series Highlighting Aggressive Features

**DOI:** 10.1002/ccr3.72151

**Published:** 2026-03-02

**Authors:** Rafael Castellanos Bueno, Gabriela Almeyda Carreño, Diana Carolina Vergara Arenas, Laura Yibeth Esteban Badillo, Ernesto García Ayala

**Affiliations:** ^1^ GERMINA‐UIS Group, Department of Internal Medicine Industrial University of Santander Santander Colombia; ^2^ GERMINA‐UIS Group, PAT UIS Group Industrial University of Santander Santander Colombia; ^3^ Cardiovascular Foundation of Colombia Santander Colombia; ^4^ PAT UIS Group, Department of Pathology Industrial University of Santander Santander Colombia

**Keywords:** clinical, immunohistochemistry, lymphatic metastasis, neoplasm invasiveness, papillary, pathology, solid subtype, solid variant, thyroid cancer, thyroid neoplasms

## Abstract

Papillary thyroid carcinoma (PTC) is the most common malignant thyroid neoplasm. Its solid/trabecular subtype (STPTC) is rare, and its aggressiveness remains controversial. We describe the clinical, histopathological, and immunohistochemical characteristics of three STPTC cases, highlighting their biological behavior and impact on current classification. Two patients exhibited aggressive clinical courses with extensive lymphatic metastases and possible distant spread, while the third case showed a more indolent progression. Immunohistochemistry revealed positivity for cytokeratin 7, cytokeratin 19, thyroglobulin, TTF‐1, and PAX8, with negativity for CD56, synaptophysin, and p63, among others. Although the 2022 WHO classification does not consider STPTC an aggressive subtype, the literature and our findings suggest reconsideration. Immunohistochemistry and the Ki‐67 proliferation index may be key tools for differential diagnosis and prognosis. STPTC can exhibit aggressive behavior, particularly in young patients. Early diagnosis and individualized therapeutic approaches are essential to improve clinical outcomes.

## Key Clinical Message

The solid subtype of papillary thyroid carcinoma may exhibit aggressive clinical behavior, particularly in young patients, with extensive lymph nodes and distant metastases. Recognizing this pattern is essential for accurate diagnosis and for guiding personalized treatment and follow‐up, despite its current non‐aggressive classification in the WHO guidelines.

## Graphical abstract

Hematoxylin and eosin (H&E) stained section, 40×. Tumor cells with “Orphan Annie eye” nuclei arranged in solid nests.

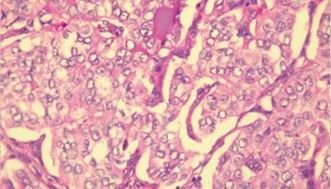

## 1 Introduction

Thyroid nodules affect approximately 20% of the population, of which about 95% are benign [1]. Among malignant cases, thyroid cancer is the most common endocrine neoplasm [2]. In the United States, it ranks as the fifth most frequently diagnosed cancer in women and ninth in men, with a continuous rise in incidence over the past 30 years [2, 3]. Globally, in 2020, its incidence was 10.1 per 100,000 women and 3.1 per 100,000 men, with mortality rates of 0.5 and 0.3 per 100,000, respectively [4]. In Colombia that same year, the incidence was 9.1 per 100,000 inhabitants, making it the fourth most common malignant neoplasm in women [5].

Tumor staging is performed using the TNM system from the American Joint Committee on Cancer (AJCC) and the International Union Against Cancer (UICC), which assesses T (primary tumor), N (regional lymph nodes), and M (distant metastases), with numeric indices reflecting disease progression [6].

Histologically, most thyroid carcinomas arise from epithelial cells and are often well‐differentiated. Papillary thyroid carcinoma (PTC) is the most common type, accounting for 70%–90% of cases [7]. It can occur at any age and predominantly affects women, with a 3:1 female‐to‐male ratio [8]. The fifth edition of the World Health Organization (WHO) histological classification of thyroid tumors, published in 2022, replaced the term “variants” with “subtypes” to avoid confusion with genetic variants, and currently recognizes 13 subtypes of PTC. Notably, the macrofollicular and multinodular subtypes are no longer included [9]. The most common subtype is the formerly termed follicular variant, characterized by predominant follicular architecture [10]. The aggressive histological subtypes include tall cell, columnar cell, and hobnail PTC, which typically present at an older age, often with angioinvasion, advanced pathological stage, and higher mitotic activity [9].

The solid/trabecular subtype of PTC (ST‐PTC), previously known as the solid variant, is rare and accounts for 1%–3% of all PTC subtypes [10]. It is an uncommon and poorly characterized form, with vascular invasion and extrathyroidal extension reported in approximately one‐third of cases [11], and a higher frequency of distant metastases [12]. For diagnosis, more than 50% of the tumor mass must exhibit a solid, trabecular, or nested growth pattern [9, 13]. Compared to classical PTC, ST‐PTC carries a less favorable prognosis [12], with a 10‐year survival rate of 90% [14]. It is essential to distinguish ST‐PTC from high‐grade aggressive follicular‐derived thyroid carcinomas, as the former typically lacks tumor necrosis and high mitotic activity.

An aggressive clinical course has been reported in both the diffuse sclerosing and solid/trabecular subtypes of PTC in the current and past literature [12, 15, 16, 17]. However, the most recent WHO classification does not categorize these subtypes as aggressive [9, 13, 15, 18]. Given the lack of definitive evidence on the aggressive nature of ST‐PTC, the objective of this report is to present the clinical and histopathological characteristics of three patients with this histological subtype, two of whom exhibited an aggressive disease course.

To ensure consistency in case selection, we retrospectively reviewed all thyroid carcinoma diagnoses recorded in the pathology archives of our institution between 2016 and 2024. We included only cases that met the histopathological criteria for papillary thyroid carcinoma and exhibited a solid, trabecular, or nested growth pattern involving more than 50% of the tumor mass, in accordance with the 2022 WHO classification. Clinical, radiological, histopathological, and immunohistochemical data were extracted from medical records and pathology reports. No additional exclusion criteria were applied.

## 2 Case Presentation

### 2.1 Case 1

A 12‐year‐old male with a history of cervical lymphadenopathy and a previous negative biopsy for malignancy in 2011 at another institution. Six years later, he presented again due to recurrence of the lymphadenopathy, associated with constitutional symptoms. On physical examination, he had an increased neck diameter and bilateral cervical lymph node conglomerates with painful adenopathies up to 4 × 5 cm in size. The thyroid gland was palpable and of normal consistency. No information regarding family medical history or psychosocial background was available beyond what is described here.

A biopsy of the right cervical lymph node (06/10/2017) revealed malignant epithelial tumor cells arranged in papillary structures with fibroconnective cores, focally involving the capsule. The cells were cuboidal with scant cytoplasm, oval pleomorphic nuclei, nuclear grooves, chromatin margination, and intranuclear inclusions. The diagnosis was metastatic classical papillary thyroid carcinoma involving a 2.7 × 2.0 × 1.3 cm lymph node, with focal capsular invasion.

**FIGURE 1:**
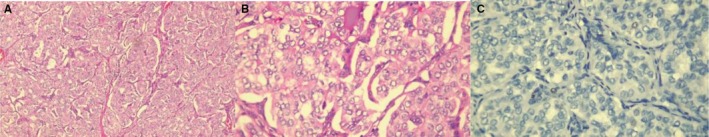
(A) Hematoxylin and eosin (H&E) stained section, 10×. Solid papillary tumor with organoid appearance. (B) H&E stain, 40×. Tumor cells show uniformly clear “Orphan Annie eye” nuclei with peripheral chromatin condensation. (C) Ki‐67 proliferation index, 40×, estimated at 5%.

He underwent total thyroidectomy with lymph node dissection (27/10/2017), preceded by thyroid hormone replacement. Three weeks later, he developed tumor recurrence in the surgical bed with four cervical nodules and underwent bilateral modified radical neck dissection with ligation of cervical vessels. The final pathology revealed an enlarged thyroid gland weighing 129 g. A predominantly solid, tan‐golden lesion with a fleshy surface measured 2.1 × 1.6 cm. Microscopic examination showed malignant epithelial cells with cuboidal morphology, clear glassy nuclei (Orphan Annie eye), nuclear grooves, and intranuclear inclusions, arranged in solid nests with frequent psammoma bodies. The tumor infiltrated surrounding tissue with moderate focal desmoplastic reaction, involved the capsule, and extended into perithyroidal soft tissue (Figure 1A,B). Chest CT showed a 2.5 mm subpleural nodule in the right lung and a 4 mm nodule in the anterior segment of the upper lobe, whose metastatic nature could not be confirmed by complementary studies (Tg/scan).

The diagnosis of ST‐PTC was supported by a solid growth pattern comprising > 50% of the tumor together with classic nuclear features of PTC. Poorly differentiated thyroid carcinoma was excluded due to the absence of tumor necrosis and low mitotic activity (Ki‐67 ≤ 5%). Medullary thyroid carcinoma was ruled out through immunohistochemistry showing strong positivity for thyroglobulin, CK7, CK19, TTF‐1 and PAX8, and negativity for calcitonin, synaptophysin, and chromogranin. This profile confirmed follicular epithelial origin and supported the diagnosis of ST‐PTC according to WHO 2022 criteria.

**FIGURE 2:**
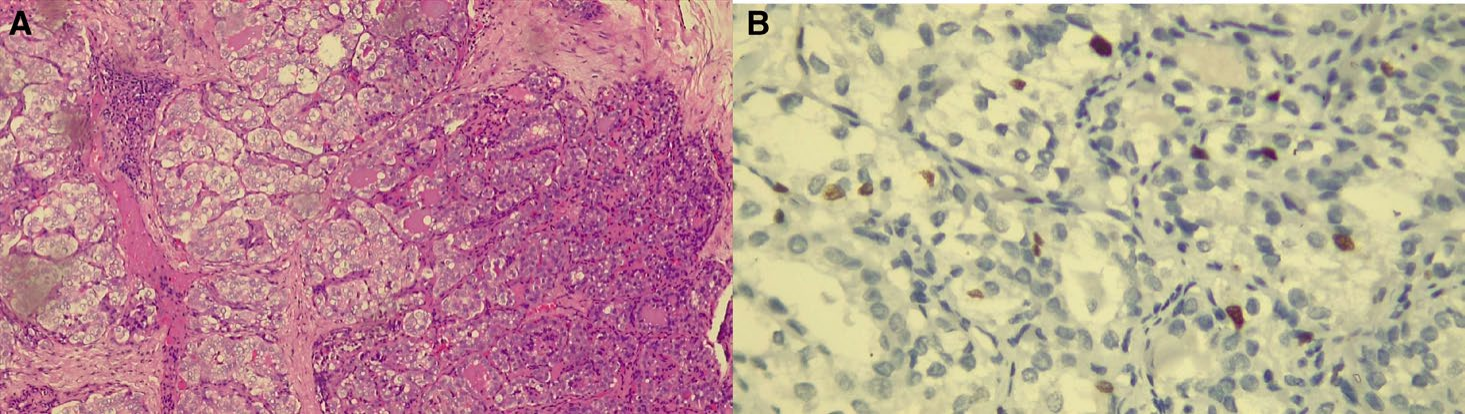
(A) H&E stain, 10×. Solid round nests completely filled with follicles. Tumor cells show “Orphan Annie eye” nuclei. (B) Ki‐67 proliferation index, 40×. < 5%.

**FIGURE 3:**
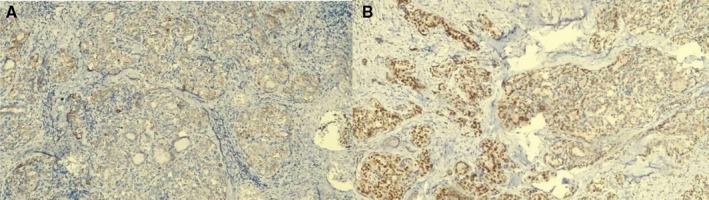
Immunohistochemical findings—positive markers. (A) Cytokeratin 19, 10×. (B) TTF‐1, 10×.

**FIGURE 4:**
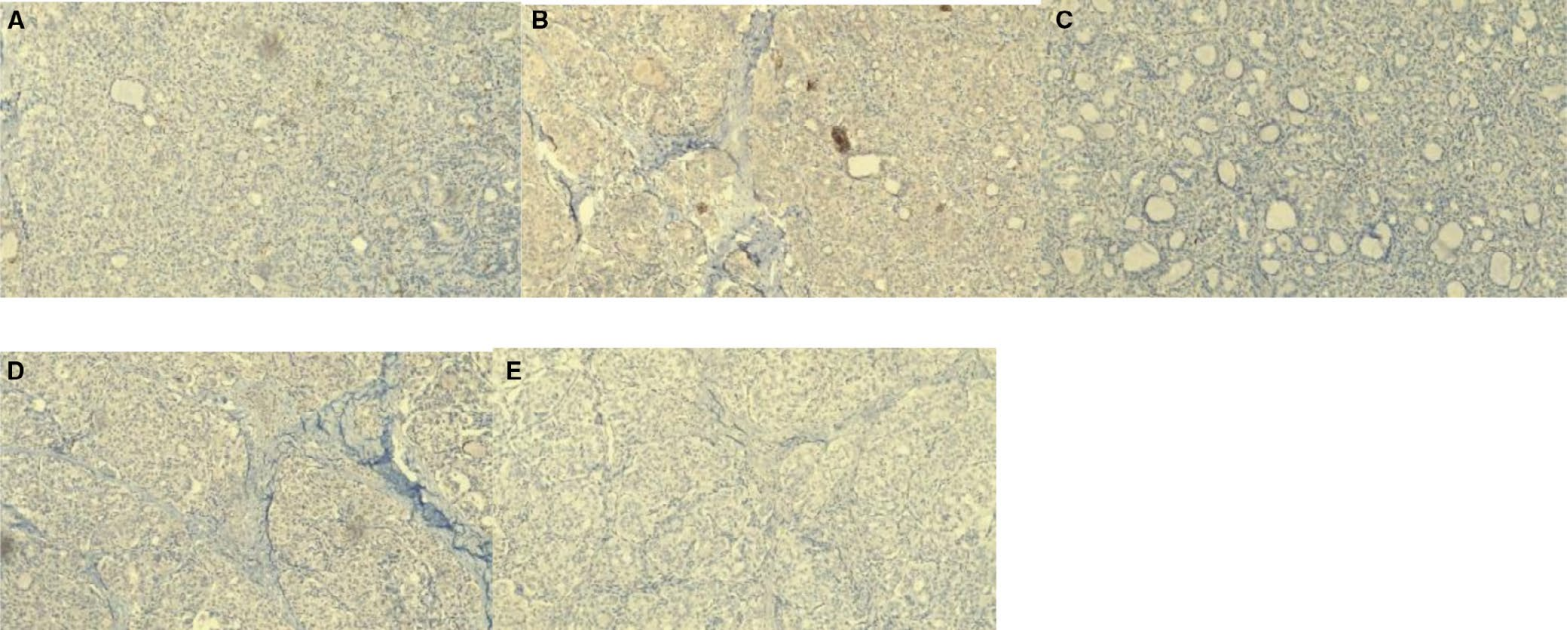
Immunohistochemical findings—negative markers. (A) CD56, 10×. (B) Calcitonin, 10×. (C) Synaptophysin, 10×. (D) Chromogranin, 10×. (E) P63, 10×.

Final diagnosis: multicentric solid subtype papillary thyroid carcinoma (2.1 × 1.6 cm), with capsular invasion, lymphovascular invasion, and massive regional lymph node metastasis, likely with pulmonary metastasis (Figures 2, 3, 4). Given that the patient is under 55 years of age, the presence of distant metastasis (M1) classifies the tumor as Stage II. Specific levothyroxine dosing/TSH suppression goals, details regarding Radioactive Iodine (RAI) therapy, and postoperative serum thyroglobulin trends are unavailable.

### 2.2 Case 2

A 40‐year‐old female previously treated with radioactive iodine for hyperthyroidism associated with grade III goiter. Due to persistence of symptoms, she underwent total thyroidectomy in 2015. Intraoperative findings included an enlarged, slightly multinodular thyroid gland with a dominant brown‐yellowish nodule in the left lobe measuring 2.1 × 1.2 cm. No additional family history or psychosocial information was available for this case.

Histological examination with hematoxylin and eosin staining (Figure 2A) revealed a malignant epithelial tumor composed of cuboidal cells with moderate cytoplasm, glassy nuclei, mild pleomorphism, nuclear grooves, and intranuclear inclusions. The cells were arranged in small cohesive nests in a solid pattern separated by thin fibrous septa. The lesion was well‐demarcated and focally involved the thyroid capsule, with moderate focal desmoplastic response and no vascular invasion.

The solid subtype of PTC was identified based on a predominantly solid architecture (> 50%) and typical nuclear features of PTC. Poorly differentiated thyroid carcinoma was excluded due to the absence of necrosis, low mitotic rate, and Ki‐67 ≤ 5%. Medullary thyroid carcinoma was excluded by negative neuroendocrine markers and strong positivity for thyroglobulin, TTF‐1, CK19, and PAX8, consistent with follicular cell lineage. Other subtypes of PTC were excluded due to lack of their defining cytological features.

Final histopathological diagnosis: solid subtype papillary thyroid carcinoma (Figures 2, 3, 4). Given that the patient is under 55 years of age and has no distant metastasis (M0), the staging corresponds to Stage I. Data detailing subsequent TSH suppression goals for the carcinoma, any possible adjuvant RAI for the carcinoma, and serum thyroglobulin follow‐up are currently inaccessible.

### 2.3 Case 3

A 31‐year‐old female with a history of cleft lip and palate repair. During her fourth pregnancy, a thyroid nodule was detected with normal thyroid hormone levels. On physical examination, a 4 cm firm, rubbery, mobile, non‐tender nodule was palpated in the left lobe. A thyroid ultrasound (24/08/2013) revealed a right lobe measuring 3.8 × 1.3 × 1.2 cm (3.3 cc), and the left lobe was almost entirely occupied by a mixed nodule (predominantly solid with microcysts) measuring 4.3 × 3.0 × 2.1 cm (14 cc), with no lymphadenopathy. A second ultrasound (12/02/2014) described a predominantly solid, hypoechoic nodule in the left lobe with benign appearance (ATA low‐risk), measuring 3 × 2 × 4 cm and surrounded by a hypoechoic peripheral halo; the right lobe was normal. No family medical history or psychosocial data could be retrieved for this patient beyond what is included here.

In November 2014, she was evaluated by endocrinology with a fine needle aspiration (FNA) result categorized as Bethesda V, suspicious for papillary neoplasm. Thyroid function tests remained normal. On 20/05/2016, she underwent total thyroidectomy. The left thyroid lobe contained a mixed solid‐cystic tumor with a reddish and vascularized outer surface, measuring 4.5 × 4.2 × 3 cm. On sectioning, the lesion displayed a heterogeneous surface with brownish‐yellow solid areas and cystic degeneration. Frozen section suggested small, hyperplastic follicles lined by cuboidal epithelium without atypia, along with fibrosis, hemorrhage, edema, vascular congestion, and mild mononuclear infiltrate—considered inconclusive.

Paraffin sections revealed multinodular goiter with a 1 × 1 cm malignant epithelial lesion composed of intermediate‐sized cells with scant cytoplasm, glassy oval to round nuclei, occasional hyperchromasia, chromatin margination, and nuclear grooves. The cells formed small nests separated by thin fibrous septa in a solid pattern, without clear papillary structures. The lesion reached the external capsule without lymphovascular invasion.

The diagnosis of solid subtype PTC was based on > 50% solid architecture with classical PTC nuclear features. Poorly differentiated carcinoma was excluded due to the absence of necrosis, low mitotic index, and Ki‐67 ≤ 5%. Medullary thyroid carcinoma was ruled out by negative neuroendocrine markers and positive staining for thyroglobulin, CK7, CK19, TTF‐1, and PAX8, confirming follicular origin. Other PTC variants were excluded due to the lack of their characteristic morphology.

Final diagnosis: non‐encapsulated solid subtype papillary thyroid carcinoma (1.2 × 1 cm). Given that the patient is under 55 years of age and no M1 disease is reported, the staging corresponds to Stage I. The patient subsequently received 30 mCi of radioactive iodine but the specific administration date, levothyroxine dose/TSH suppression targets, and long‐term thyroglobulin trends are not documented.

**FIGURE 5:**
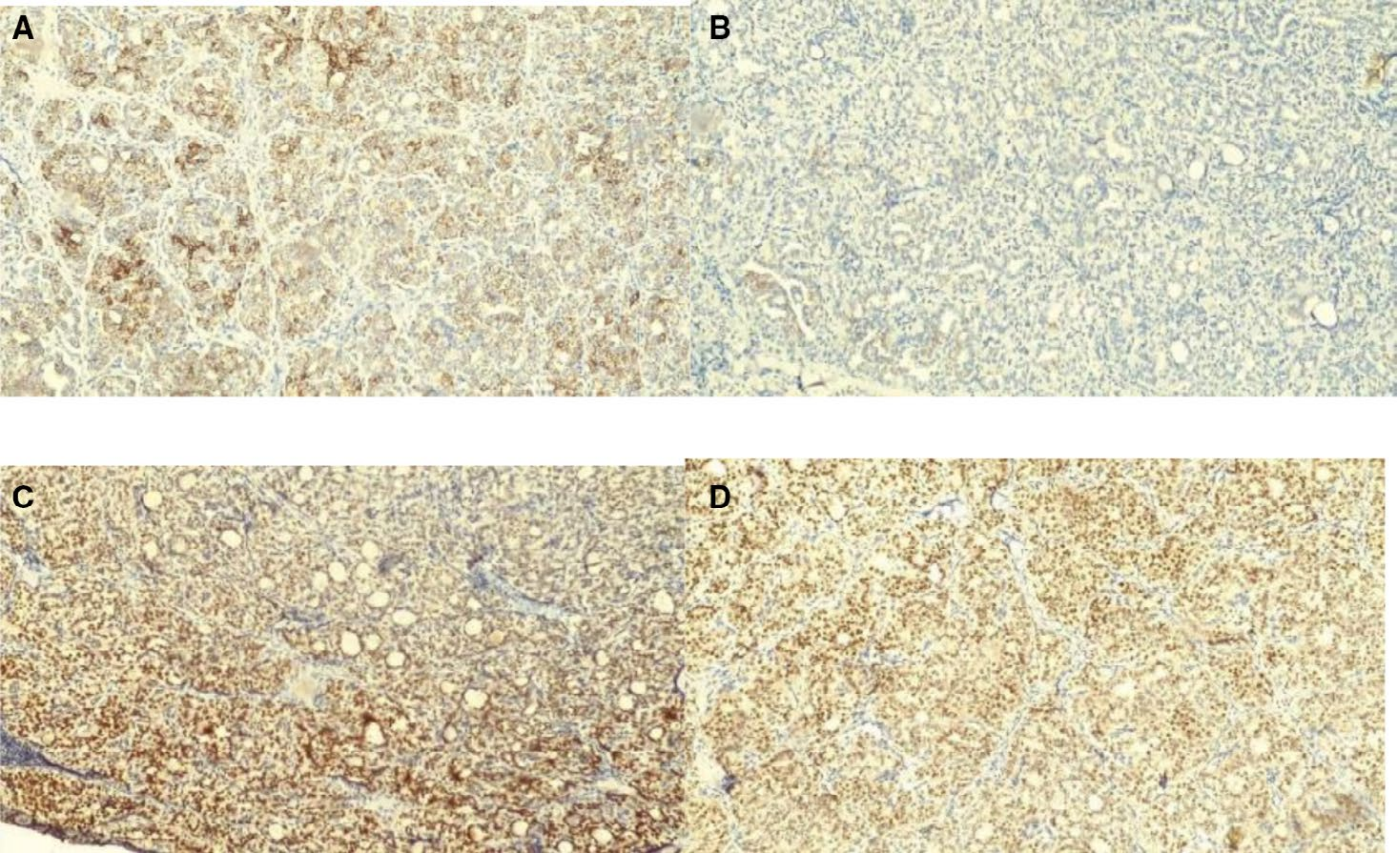
Immunohistochemical findings—positive markers. (A) Cytokeratin 7, 10×. Strongly positive. (B) Cytokeratin 19, 10×. Positive (50%). (C) TTF‐1, 10×. Strongly positive. (D) Pax8, 10×. Positive.

**FIGURE 6:**
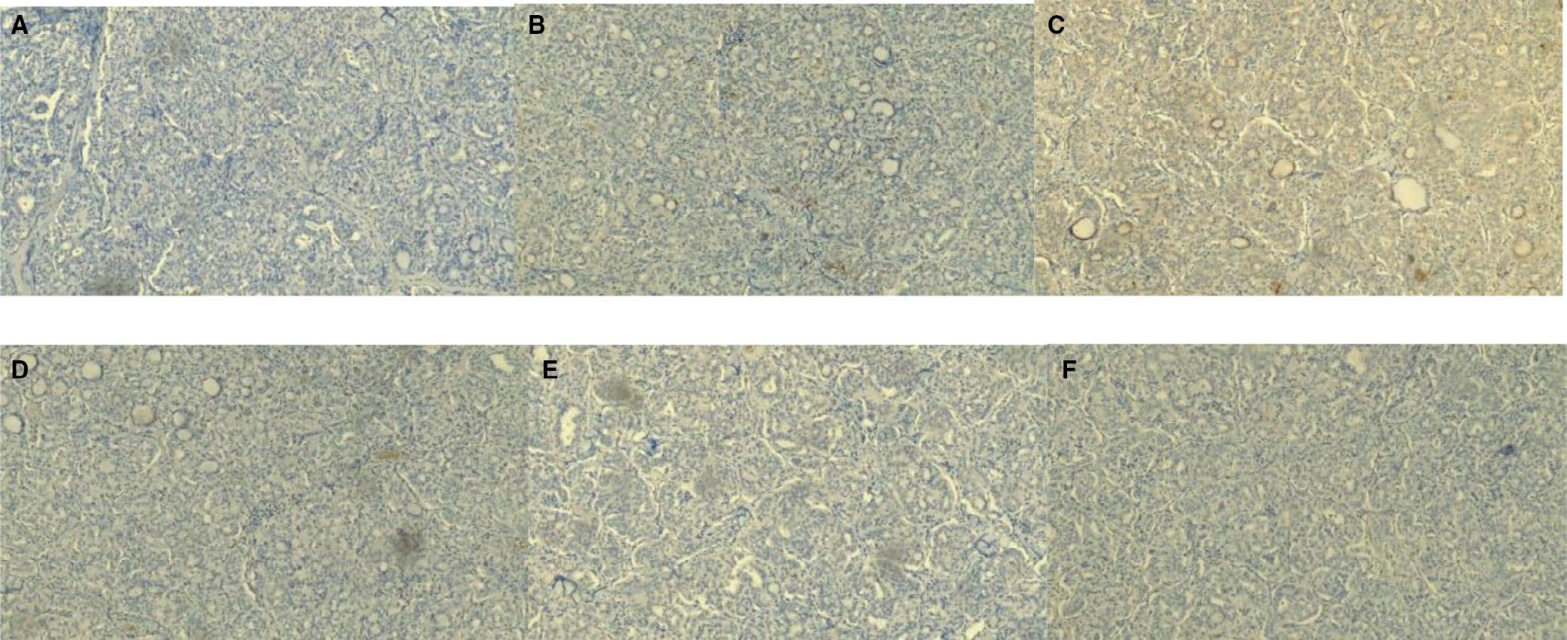
Immunohistochemical findings—negative markers. (A) Cytokeratin 20, 10×. (B) CD56, 10×. (C) Calcitonin, 10×. (D) Synaptophysin, 10×. (E) Chromogranin, 10×. (F) P63, 10×.

All three cases underwent routine paraffin‐embedded histopathological processing with hematoxylin and eosin staining (Figures 1A,B and 2A). Subsequently, immunohistochemical staining was performed, revealing strong positivity for cytokeratin 7, cytokeratin 19, thyroglobulin, TTF‐1, and PAX8 (Figure 5), and negativity for CD56, synaptophysin, and p63, among others (Figure 6). These results are summarized in Table 1.

**TABLE 1:** Immunohistochemical markers.

Marker	Case #1	Case #2	Case #3
Cytokeratin 7	Strongly positive	Strongly positive	Strongly positive
Cytokeratin 19	Positive (50%)	Positive (70%–80%)	Positive (40%)
Cytokeratin 20	Negative	Negative	Negative
CD56	Negative	Negative	Negative
Thyroglobulin	Strongly positive	Strongly positive	Strongly positive
Galectin	Strongly positive	Strongly positive	Strongly positive
Calcitonin	Negative	Negative	Negative
Ki‐67	5%	< 5%	5%
Synaptophysin	Negative	Negative	Negative
Chromogranin	Negative	Negative	Negative
P63	Negative	Negative	Negative
TTF‐1	Strongly positive	Strongly positive	Strongly positive
Pax8	Positive	Positive	Positive

## 3 Discussion

The solid subtype of papillary thyroid carcinoma (ST‐PTC) has been described as more frequent in children, particularly those with a history of radiation exposure, although it is not limited to this group. This is exemplified by our first case: a child with no history of radiation who presented with extensive lymph node involvement and probable pulmonary metastases. This aligns with current evidence indicating that pediatric PTCs tend to present with larger primary tumors, more regional lymph node metastases, and a higher rate of distant disease at diagnosis compared to adult cases [9, 13, 15, 18]. Moreover, children are at greater risk of pulmonary and regional lymphatic metastases [9, 13, 15, 18].

In the aforementioned case, the initial pathology report diagnosed classical PTC. It was only upon further review that the diagnosis of the solid subtype was established, consistent with previous reports such as that of a 12‐year‐old girl with cervical lymphadenopathy and no radiation exposure, whose fine‐needle aspiration cytology suggested medullary thyroid carcinoma versus Hürthle cell neoplasm, requiring histopathological analysis for definitive diagnosis [19].

Thyroid cancer typically presents as an asymptomatic thyroid nodule, with diagnosis confirmed by fine‐needle aspiration (FNA). Most cases of PTC are diagnosed based on classical histopathological features seen on hematoxylin–eosin (H&E) staining. ST‐PTC is defined by a solid, trabecular, or nested growth pattern comprising more than 50% of the tumor volume, with rounded or oval solid nests resembling colloid‐filled thyroid follicles [9]. A crucial diagnostic criterion is the presence of characteristic nuclear features such as clear “Orphan Annie eye” nuclei. This variant is believed to develop when proliferation exceeds secretion [20]—features observed in our cases.

The main differential diagnosis on H&E staining is poorly differentiated thyroid carcinoma, which may share similar architecture but lacks the nuclear features typical of PTC [20].

In cases with ambiguous morphology, immunohistochemical markers are essential for diagnosis. CK19 typically shows strong positivity, while CD56 is negative in ST‐PTC. P63 has shown limited diagnostic utility. Bolívar et al. analyzed 29 cases of PTC and found 100% strong and diffuse CK19 positivity, 88% CD56 negativity, and only 64% focal nuclear P63 positivity [21]. Similarly, Unger et al. demonstrated P63 expression in a high proportion of PTCs and Hashimoto's thyroiditis, suggesting it may help differentiate PTC from other thyroid neoplasms—although its absence does not exclude the diagnosis [22].

In all three cases presented, immunohistochemical analysis revealed strong CK7 and CK19 positivity, as well as positive staining for thyroglobulin, TTF‐1, galectin, and PAX8. Conversely, CK20, P63, CD56, synaptophysin, and chromogranin were negative. Despite the aggressive course observed in some cases, the Ki‐67 proliferation index was 5% in two cases and below 5% in the third. Ki‐67 is a useful marker for cell proliferation; an elevated index (> 5%) is often seen in infiltrative tumors [23]. Hence, a high Ki‐67 index can aid in diagnosing aggressive thyroid carcinomas and predicting prognosis [23, 24]. However, not all morphologically aggressive variants exhibit a high index. Recent literature notes that aggressive PTC variants with an indolent clinical course may occur in young patients, are often encapsulated or well circumscribed, show low mitotic activity, and—most importantly—have a low Ki‐67 index [9]. This suggests that proliferative activity in primary lesions is not necessarily linked to metastatic spread or the growth of nodal metastases; Case 1, with a Ki‐67 ≤ 5% despite an aggressive clinical course, illustrates this dissociation [15, 25]. For a concise comparison of the key immunohistochemical findings used in the differential diagnosis (Table 2).

**TABLE 2:** Differential diagnosis of ST‐PTC, medullary, and high‐grade follicular‐derived thyroid carcinomas.

Cell origin	Follicular markers (Tg, TTF‐1, PAX8)	Neuroendocrine markers (calcitonin, chromogranin, synaptophysin)	CK19	CD56	High‐grade features (necrosis/Ki‐67)	
ST‐PTC (solid/trabecular subtype of papillary thyroid carcinoma)	Follicular epithelial	Positive (thyroglobulin, TTF‐1, PAX8)	Negative (calcitonin, chromogranin, synaptophysin)	Positive (strong, typically)	Negative	Lacks tumor necrosis or high mitotic rate. Ki‐67 index typically ≤ 5%
MTC (medullary thyroid carcinoma)	Parafollicular C‐cell	Negative for follicular markers (Tg, TTF‐1, PAX8)	Positive (calcitonin, chromogranin, synaptophysin)	Variable/NA	Often positive/NA	High Grade defined by ≥ 5 mitoses per 2 mm^2^, necrosis, and/or Ki‐67 ≥ 5%
DHGTC/PDTC (differentiated high‐grade/poorly differentiated carcinoma)	Follicular‐derived	Positive (maintains TTF‐1, PAX8, Tg positivity)	Negative	Often positive	Negative/NA	Required: Tumor necrosis and/or high mitotic rate (≥ 5 mitoses per 2 mm^2^); Ki‐67 typically 10%–30% or more

*Source:* Adapted from Jung et al. [9] and Baloch et al. [15], WHO classification of thyroid tumors.

Preoperative subtyping of PTC can help refine treatment strategies and guide more precise surgical interventions. Molecular testing of FNA cytology is increasingly common, particularly because BRAF mutations are highly prevalent and specific to PTC, and not found in benign thyroid lesions [26, 27]. In ST‐PTC, a unique BRAF gene triplet suppression involving the substitution of valine and lysine by glutamate (BRAF V600E + K601) has been reported [28]. In a study of 500 PTC cases, Cristiana Lupi et al. identified a VK600‐1E deletion (BRAF VK600‐1E) in one case of ST‐PTC, suggesting possible genotype–phenotype correlations [29]. Unfortunately, genetic analysis could not be performed in our cases due to institutional ethics committee restrictions.

Historically, ST‐PTC has undergone evolving recognition. In 2001, Nikiforov et al. analyzed 20 ST‐PTC cases over 27 years, reporting a predominant solid growth pattern (> 70%), classical nuclear features, and absence of tumor necrosis. They concluded that the solid subtype had a slightly higher frequency of distant metastases and a less favorable prognosis than classical PTC [30]. In 2017, a retrospective study of 27 patients with ST‐PTC found that regardless of the proportion of solid component, the subtype was associated with larger tumors, extracapsular extension, high recurrence rates, and shorter disease‐free survival compared to well‐differentiated PTC [31]. A 2018 meta‐analysis of 11 studies with 205 ST‐PTC cases showed increased risks of vascular invasion, recurrence, and cancer‐specific mortality compared to classical PTC [32].

This evidence underscores the importance of not only surgical and adjuvant treatment but also individualized follow‐up in patients with ST‐PTC. Thyroglobulin, a thyroid‐specific protein, serves as a crucial predictor of persistent or recurrent disease; levels > 10–30 ng/mL are associated with poorer survival [33]. In a retrospective study with 24‐month follow‐up, 14 ST‐PTC cases (0.23% of 6052 PTCs) exhibited extrathyroidal extension in 50% and lymph node metastases in 35.7%. None showed recurrence on postoperative serum thyroglobulin surveillance, suggesting a potentially less aggressive course [34]. Nevertheless, thyroglobulin levels may remain low even in the presence of metastases in ST‐PTC, limiting its utility as a sole follow‐up marker [35].

The cases presented herein illustrate that ST‐PTC can exhibit an aggressive clinical course, with high metastatic rates and a less favorable prognosis [12]. Despite the WHO 5th edition not classifying it as an aggressive histological subtype [9, 13, 15, 18], the growing body of literature and our clinical findings support reconsideration of its classification—especially if complemented by molecular studies [10].

## 4 Conclusions

The solid subtype of papillary thyroid carcinoma (ST‐PTC) is rare and associated with a slightly less favorable prognosis than classical variants. Although it does not typically present the aggressive clinical behavior of poorly differentiated thyroid carcinomas, its elevated risk of recurrence and distant metastasis—especially in pediatric patients—warrants a high index of suspicion. Once diagnosed, an aggressive treatment plan based on appropriate surgery, radioactive iodine therapy, and thorough follow‐up is essential to improve outcomes.

## Author Contributions

**Rafael Castellanos Bueno:** supervision, validation, writing – review and editing. **Gabriela Almeyda Carreño:** conceptualization, writing – original draft, writing – review and editing. **Diana Carolina Vergara Arenas:** conceptualization, writing – original draft, writing – review and editing. **Laura Yibeth Esteban Badillo:** writing – original draft. **Ernesto García Ayala:** supervision, validation, writing – review and editing.

## Funding

The authors have nothing to report.

## Consent

Written informed consent was obtained from all three patients for the publication of their clinical information and any accompanying images. The original signed consent forms were scanned and submitted as supporting information during the submission process.

## Conflicts of Interest

The authors declare no conflicts of interest.

## Data Availability

The data supporting the findings of this study are not publicly available due to patient privacy and ethical restrictions but are available from the corresponding author upon reasonable request.
